# A Mobile Gaming Intervention to Increase Adherence to Antiretroviral Treatment for Youth Living With HIV: Development Guided by the Information, Motivation, and Behavioral Skills Model

**DOI:** 10.2196/mhealth.8155

**Published:** 2018-04-23

**Authors:** Laura Whiteley, Larry Brown, Michelle Lally, Nicholas Heck, Jacob J van den Berg

**Affiliations:** ^1^ Department of Psychiatry Rhode Island Hospital Providence, RI United States; ^2^ Department of Psychiatry and Human Behavior Warren Alpert Medical School Brown University Providence, RI United States; ^3^ Division of Infectious Disease Veterans Administration Medical Center Providence, RI United States; ^4^ Department of Psychology Marquette University Milwaukee, WI United States; ^5^ Department of Behavioral and Social Sciences School of Public Health Brown University Providence, RI United States

**Keywords:** mobile phones, adolescents, young adults, patient compliance

## Abstract

**Background:**

Highly active combination antiretroviral treatment has been shown to markedly improve the health of HIV-infected adolescents and young adults. Adherence to antiretroviral treatment leads to decreased morbidity and mortality and decreases the number of hospitalizations. However, these clinical achievements can only occur when young persons with HIV are adherent to care. Unfortunately, adolescents and young adults have poorer rates of adherence to antiretroviral medications and poorer rates of retention in care than older adults. Novel and engaging digital approaches are needed to help adolescents and young adults living with HIV be adherent to treatment.

**Objective:**

The aim of this study was to develop an immersive, action-oriented iPhone gaming intervention to improve adherence to antiretroviral medication and treatment.

**Methods:**

Game development was guided by social learning theory, taking into consideration the perspectives of adolescents and young adults living with HIV. A total of 20 adolescents and young adults were recruited from an HIV care clinic in Rhode Island, and they participated in qualitative interviews guided by the information-motivation-behavioral skills model of behavior change. The mean age of participants was 22 years, 60% (12/20) of the participants identified as male, and 60% (12/20) of the sample reported missing a dose of antiretroviral medication in the previous week. Acceptability of the game was assessed with client service questionnaire and session evaluation form.

**Results:**

A number of themes emerged that informed game development. Adolescents and young adults living with HIV desired informational game content that included new and comprehensive details about HIV, details about HIV as it relates to doctors’ visits, and general health information. Motivational themes that emerged were the desire for enhancement of future orientation; reinforcement of positive influences from partners, parents, and friends; collaboration with health care providers; decreasing stigma; and increasing personal relevance of HIV care. Behavioral skills themes centered on self-efficacy and strategies for medical adherence and self-care. On the client service questionnaire, 10 out of the 11 participants indicated they were “satisfied with the game activities,” and 9 out of 11 “would recommend it to a friend.” On the session evaluation form, 9 out of 11 agreed that they “learned a lot from the game.”

**Conclusions:**

We utilized youth feedback, social learning theory (information-motivation-behavioral skills), and agile software development to create a multilevel, immersive, action-oriented iPhone gaming intervention to measure and improve treatment adherence for adolescents and young adults living with HIV. There is a dearth of gaming interventions for this population, and this study is a significant step in working toward the development and testing of an iPhone gaming app intervention to promote adherence to antiretroviral treatment.

**Trial Registration:**

ClinicalTrials.gov NCT01887210; http://clinicaltrials.gov/ct2/show/NCT01887210 (Archived by WebCite at http://www.webcitation.org/6xHMW0NI1)

## Introduction

### Background

According to the Centers for Disease Control and Prevention, young persons aged 13-29 years accounted for 41% of the new HIV infections in the United States in 2015 [1], and an estimated 99,463 adolescents and young adults in the United States are living with HIV/AIDS (acquired immunodeficiency syndrome) [1]. Advances in treatments for HIV can allow those infected to manage their HIV infection as a chronic, rather than imminently life-threatening, disease [2]. However, these achievements can only be made when persons living with HIV take their medications as prescribed and maintain consistent medical care [3-6]. Unfortunately, adolescents and young adults with HIV are the least likely out of any age group to be adherent to care and have a suppressed viral load [3,7].

The percentage of prescribed doses of antiretroviral medications taken by adolescents and young adults ranges from 50% to 75% in the United States [7-9], and studies, with adults, adolescents, and children, show that achieving and maintaining an extremely high level of medication adherence (approximately 90%) is needed to obtain the full benefits of antiretroviral treatment (ART) [3,10,11]. Many of the barriers to ART adherence are similar for adults and youth; however, there are additional barriers for adolescents and young adults living with HIV [12]. Developmentally, late adolescence and young adulthood is an age-related period characterized by less inhibition, increased risk-taking, decreased motivation for planning, and less parental monitoring [13,14]. Adolescents and young adults can feel invulnerable to consequences, and this can explain the risk-taking and limit-testing behaviors seen during adolescence. Often, adolescents and young adults do not have fully developed risk assessment skills, impulse control, or organizational abilities. These characteristics are believed to contribute to lower rates of adherence to care among this age group [13-15].

### The Efficacy of Digital Interventions

Digital interventions to improve adherence to ART for adolescents and young adults hold particular promise [16]. Adolescents and young adults aged 18-29 years have high rates of mobile phone use with 98% owning a mobile phone or a smartphone [17]. Technologies, such as the mobile phone, play an increasingly significant role in adolescents’ and young adults’ interpersonal and environmental life, as they communicate information, reinforce cultural norms, and influence personal identity and behaviors [18]. Furthermore, gaming is popular among youth. In the United States, 99% of teenage males and 94% of teenage females play video games, and 46% of all video gaming occurs on mobile phones or portable devices [19]. The widespread appeal of digital game playing among all adolescents and young adults creates a unique opportunity to deliver health education during leisure time, outside of the clinic, and in a manner that is cost-effective and easily scalable [20]. Games can attract and maintain attention, which is a key component for effective behavior change [21]. Compelling interactive games can expose players to essential health-related content repeatedly and also give players unlimited opportunities to rehearse new skills and receive personalized feedback on health choices made within the game [21,22]. Finally, gaming has been shown to improve motivation for healthy behaviors. Motivation, defined as the process that initiates, guides, and maintains goal-oriented actions, is key to maintaining ART adherence [20,21,23].

There is a paucity of data on gaming interventions to improve adherence to ART. However, before the widespread availability of the internet and cell phones, offline games were found (in randomized controlled trials, RCTs) to impact other health behaviors among youth living with asthma, diabetes, and cancer. A diabetes game for children, called Packy and Marlon, indicated that a well-designed, educational video game can be effective in terms of improving diabetes-related self-efficacy (*P*=.07), communication with parents about diabetes (*P*=.03), and self-care behaviors (*P*=.003). These changes occurred after diabetic youth played the game at home for 6 months, compared to a control group of diabetic youth who took home an entertainment video game that had no health content [24]. In a game called Bronkie the Bronchiasaurus, youth engage in play with a dinosaur character with asthma and help him save his homeland while trying to avoid asthma triggers (pollen, cold viruses, dust) and keep his asthma under control. An empirical study showed that playing the game for less than an hour resulted in significant improvements in a player’s asthma knowledge, self-efficacy for asthma self-management, and self-efficacy for talking with friends about asthma [25,26]. Another video game, named Re-Mission, designed for a wide age range of adolescents and young adults (13-29 years) with acute leukemia, lymphoma, and soft-tissue sarcoma showed promising effects as well. Re-Mission was designed as an action-adventure game with the main character or protagonist shooting cancer-causing agents in the bloodstream. Players gain points and strength by adhering to medications in the game fantasy world. In a randomized control study with a 3-month follow-up, 375 male and female participants who played Re-Mission had significantly improved adherence to trimethoprim-sulfamethoxazole (*P*=.01) and 6-mercaptopurine (*P*=.002) compared with controls after an average of only 10.7 hours of play. Adherence to trimethoprim-sulfamethoxazole was tracked by electronic pill monitoring devices (n=200), and the proportion of doses taken correctly by those playing Re-Mission was 19% greater than those in the control group. Self-efficacy (*P*=.01) and knowledge (*P*=.04) also increased in the Re-Mission game intervention group compared with the control group [27,28]. The successful games reviewed above provided interactive environments where players could improve motivation and engage in behavioral rehearsal [20-22,24-28].

### Gaps in Literature

Despite the promise of digital games, reviews describe that there is a paucity of published or presented abstracts related to gaming for adolescents and young adults living with HIV [16]. Hightow-Weidman et al identified 5 digital games in development to improve ART adherence (from National Institutes of Health’s RePORTER, including our intervention described here). Published outcomes or descriptions of these interventions are sparse [16]. LeGrand et al has published a description of the development phase of a game entitled Epic Allies. The game is designed to improve ART uptake, engagement in care, and adherence among young men who have sex with men (YMSM) and transgender women who have sex with men. During game play, users can earn medals and tokens for taking medications and reading health-related studies. These tokens can then be used to earn access to fun, non-HIV-related games in the app. Users can interact with one another in the app and send each other positive messages [29]. We could not find other manuscripts or published descriptions of an iPhone gaming app to improve adherence to ART for adolescents and young adults.

There are published descriptions of gaming interventions targeted to HIV negative youth, who are at risk for acquiring HIV. An evidence-based gaming intervention called PlayForward aims to reduce risk for HIV among at-risk, ethnic, and racial minority adolescents aged 11-14 years. This tablet-based game provides an interactive world using an avatar where players face challenges such as peer pressure to drink alcohol or engage in other risky sexual behaviors. Players can experience how their choices affect their health and are able to go back in time to change their choices to create different, healthier outcomes [30]. The smartphone game SwaziYolo is an interactive game for HIV-negative young adults (18-29 years) living in Swaziland, Africa. In this app, players can practice relationship and health choices while looking for love [31]. A mobile phone–optimized intervention entitled healthMpowerment is designed to reduce sexual risk behaviors among YMSM. In this intervention, YMSM can acquire *reputation points* through reading information about HIV, playing sexually transmitted infection-related games, and positive interactions with other users. These points can be used to purchase T-shirts and iPod shuffles [32].

There are no gaming interventions for older adults living with HIV. However, interventions to improve adherence to ART among older adults have tested the usefulness of less-complex technologies such as electronic reminders and/or pill bottle opening measurements. Reviews show that the most successful interventions couple these less-complex technologies with in-person interventions to improve motivation for treatment [33-35]. Measuring adherence alone (through an electronic method such as a micro-electro-mechanical systems cap) or merely reminding patients about pill taking (with an alarm) does not significantly improve adherence in the long term [35,36].

Among older adults living with HIV, there are also promising studies that have examined interactive text messaging to improve ART [37]. A meta-analysis of 8 studies, reporting 9 interventions, shows that text messaging interventions yielded significantly higher adherence than control conditions (odds ratio [OR] 1.39, 95% CI 1.18-1.64). The mean age of participants in the studies was 40 years (range  36-42 years). Sensitivity analyses of intervention characteristics suggested that studies had larger effects when intervention texts were sent less frequently than daily and included personalized communication. Text message interventions among adults were associated with improved viral load and/or CD4+ count (k=3; OR  1.56, 95% CI 1.11-2.20) [38]. Less data are available on text messaging interventions for younger populations living with HIV. Dowshen et al did report that personalized, interactive, daily short message service reminders were feasible and acceptable among youth living with HIV who were between the ages of 14 and 29 years (mean age 23 years). Participants in that study (N=25) had significantly increased self-report of adherence at 12 and 24 weeks in comparison with baseline (week 0: 74.7; week 12: 93.3, *P*<.001; week 24: 93.1, *P*<.001) [39].

Building on this knowledge, we developed a multilevel gaming intervention to improve adherence to ART for adolescents and young adults aged 18-26 years. This intervention integrates a smart pill bottle cap (that measures adherence) with an immersive iPhone game and personalized text messaging (see Multimedia Appendix 1). The gaming intervention was informed by the information-motivation-behavioral skills (IMB) theory of learning [40-42]. The iPhone gaming app was designed for participants to experience absorbing action-oriented adventures that increase information about their health (eg, knowledge about HIV treatment, transmission, adherence), improve motivation (eg, action figures experience health benefits of adherence), and build skills (interact with clinicians at appointments, take medications as prescribed). Adherence (measured by the smart pill bottle cap) and game-related text messages are integrated into the gaming intervention. This multilevel approach integrates sound theoretical principles with novel, but intuitive, technology. The aim of this paper is to describe the development of this multilevel iPhone gaming app entitled Battle Viro.

## Methods

### Gaming App Development

Development of Battle Viro was accomplished using iterative and collaborative procedures to fully integrate the clinical experiences of adolescents and young adults living with HIV, academic researchers, and technology partners. Game development was guided by qualitative interviews with a diverse group of adolescents and young adults living with HIV between the ages of 18 and 26 years. Guided by the principles of agile software development [43], the qualitative interviews and the game/app programming were synergistic. Agile software development aims for continuous design testing and adaptation based on continuous feedback [43].

As discussed in the Introduction, the adherence gaming app, Battle Viro, was designed to be consistent with the IMB model of health [44]. The IMB model is a well-established conceptualization for improving adherence to antiretroviral medication and engagement in treatment. Nongaming interventions based on IMB have demonstrated efficacy [40,41,45]. Reviews have suggested that interventions guided by accepted theories of change are more efficacious than those not driven by theory [46]. According to the IMB model, health information, motivation, and behavioral skills are the fundamental determinants of health behavior. Information that is directly relevant to adherence and HIV transmission and easily applied to an individual’s cultural and social setting is a prerequisite for success. Motivation to engage in HIV preventive behavior, including personal motivation (favorable attitudes toward adherence) and social motivation (perceived social and cultural support for performing these acts), is also essential for healthy behavior. Finally, skills for performing adherence behaviors and a sense of self-efficacy are critical components. The IMB model, consistent with social learning theory, is broadly applicable and can be used to guide game development and create theoretically consistent gaming content [46,47]. Multiple reviews have demonstrated that behavioral interventions shown to be most efficacious are those tailored for the target population and preceded by formative research to inform intervention development [48-50].

### Sample and Recruitment

Males and females, 14 to 26 years old, were eligible for enrollment in the study according to the following criteria: (1) English-speaking, (2) in medical care for HIV and receiving ART, (3) aware of their HIV status as per clinician and clinical record, (4) able to give consent/assent and not impaired by cognitive or medical limitations as per clinical assessment, and (5) adolescent assent and consent of a parent/legal guardian if under 18 years of age or consent of youth if 18 years of age or older. Those who did not meet the above-mentioned inclusion criteria were excluded.

We recruited 20 adolescents and young adults living with HIV for qualitative interviews to guide game development after institutional review board’s approval. Subjects were recruited from a convenience sample in the HIV care clinic in Rhode Island. Subjects were approached by research staff with an institutional review board-approved flyer, and written consent was obtained upon meeting with study staff for the qualitative interview. Overall, 20 subjects were approached over the course of the interviews, and all of them consented and completed the interview. Subjects were recruited until data saturation was achieved and a relative balance in the sample was achieved based on gender, age (<22 vs ≥22), race, and sexual orientation. We were not able to recruit participants younger than 18 years (as originally planned), as the vast majority of patients in our state who are diagnosed and living with HIV are older adolescents and young adults. The mean age of participants was 22 years (range 18-26 years; 8 out of the 20 were older than 22 years). Of the total participants, 60% (6/12) identified as male, and 60% (12/20) completed 12th grade. Of the participants who identified as African American (10/20, 50%), 10% (2/20) identified as Hispanic and 30% (6/20) identified as white. In total, 40% (8/20) identified as heterosexual, 40% (8/20) identified as homosexual, and 20% (4/20) identified as bisexual. Out of these 20 participants, 12 (60%) reported missing a dose of antiretroviral medication in the previous week.

### Adaptation of the Information-Motivation-Behavioral Skills Adherence Gaming Intervention

A preliminary storyboard for the IMB gaming app proposed, entitled Battle Viro, was drafted based on the popular Mission Critical Studios game entitled Dr. Nano X: Incredible Voyage Inside the Body [51]. Dr. Nano X is a 5-star-rated mobile game (the highest rating possible) in the iTunes app store and is available on both Android and iPhone. We worked directly with the development team at Mission Critical Studios to develop Battle Viro using Dr. Nano X as a framework. Adapting our game to promote ART adherence from an already existing game greatly decreased the cost of the project. Characters, actions, and IMB messaging were built specifically for Battle Viro; however, we were able to reuse backgrounds, mechanisms of game play/controls, and many sound effects from Dr. Nano X. The adapted gaming app for this project, Battle Viro, takes place inside the human body similar to Dr. Nano.

An initial storyboard was developed for Battle Viro. The storyboard starts with a short narrative movie that explains that the player is becoming miniaturized in order to enter his or her body and destroy attacking viruses and infections (see Multimedia Appendix 2). Weapons and tools that help the player destroy virus and infections in the body can be earned by taking medications and building an alliance with medical staff. The first level begins on the surface of the skin. If players successfully battle virus, engage with providers, take medication, and make healthy decisions, they move to the next exciting level. As players become expert nanobots, they move onward through the arterial system, the lungs, kidneys, brain, and other new, vibrant, and distinctive organ systems (see Multimedia Appendices 3-5). Throughout Battle Viro, multiple messages from doctors, clinicians, and friends are integrated into play as the nanobot/protagonist successfully destroys virus and other opponents (ie, opportunistic infections) (see Multimedia Appendix 6). At the end of each level or each mission, the player’s score (health status and pill count) is shown (see Multimedia Appendix 7). Each level provides new challenges in colorful body organs; however, the mission stays the same: kill the virus and build strength through taking medicine, learning information, improving motivation, and engaging with healthy characters to build skills.

### Interview Topics

The interview guide consisted of focused, but open-ended, questions aimed at maximizing participant responses (see Textbox 1). Participants were asked about information relevant to medical adherence, motivating factors to adherence, and behavioral skills needed for adherence. The IMB model guided interview content, and participants were also queried about their general gaming experience and their reactions to the storyboard and gaming content. The qualitative interviews and app programming were synergistic [43]. Therefore, 11 participants were shown storyboards of the game and feedback was elicited, and additional 9 participants were shown an interactive iPhone version of the game as it became available, and feedback was elicited to inform further game design and development.

### Information Needed for Adherence

Participants were asked about knowledge and information that have influenced their adherence to medication and engagement in medical appointments. Questions included “What type of information from doctors or friends makes it easier to take medications for HIV?” and “What information makes it easier to come to appointments?” This part of the interview aimed to understand the specific knowledge about HIV and ART that promotes adherence behaviors. For example, probes focused on how appropriate administration, expected side effects, and drug interactions can influence adherence to medication and care (for more examples, see Textbox 1).

**Qualitative interview guide based on the information-motivation-behavioral skills model.**

Questions and probes

Information

- Was there knowledge or information that helped you at different times or at different ages (older vs younger)?
- Does different knowledge or information about HIV and medication help boys vs girls?
- What knowledge about antiretroviral treatment promotes adherence to meds?
- Does knowing about side effects and drug interactions change decision making to take medications?

Motivation

- What are the main issues in medical care for HIV?
- What are the things that make it hard to take HIV medications?
- What are the attitudes or feelings that teens like you have that make it harder to take meds? Or easier to take meds?
- How do partners, your family, and your community play a role in adherence to care?

Behavioral skills

- Do you use alarms, your phone, or reminders?
- What do you do if you miss a dose of medication?
- What are the strategies for adherence over time and across different situations?
- Are there things that you do such as eating, or avoiding certain substances, that make taking medication easier?

General gaming attitudes

- What is your reaction to getting some HIV information and skills in a game?
- Do you ever play games that teach you facts or in which you learn something?
- Do you go online or use your phone to learn information about your health?
- Have you ever played a health-related game before on your phone or at a computer?

Reactions to Battle Viro

- What did you like and not like about it?
- What do you think this activity is trying to teach you?
- How much did the material look like the other games that you play?
- How could this activity or content be improved for teens your age?
- Now that you have seen this game, would you want to play it?
- Before you came here today, did you ever find anything like this in a game on a phone or on a computer?
- Would you be worried about playing the games when others could see it?
- What would you say if someone asked you about the game?

### Motivation for Adherence

Participants were queried about motivational issues related to adherence with probes such as “I would like to hear about what you think the serious issues are surrounding taking HIV medications and coming to medical appointments” and “What are the things that make it hard to take HIV medications?” This part of the interview was dedicated to understanding both personal and social motivations for adherence. Queries were focused on the positive and negative attitudes toward taking antiretroviral medications, perceived negative effects of nonadherence, and the individual’s perceptions of social support from significant others, family, friends, and medical care providers (for more examples, see Textbox 1).

### Behavioral Skills for Adherence

Participants were asked about the behavioral skills needed for adherence. Participants were also asked about their ability to perform necessary adherence-related tasks and his/her perceived self-efficacy for these tasks. Questions included “What are the ways that you remember to take medications and remember your appointments?” and “What events in your life make it harder to remember to take medication? Or remember your appointments?” We also asked participants about strategies for self-reinforcement for adherence over time and across different situations. We asked questions such as “Do you consciously think about your medication schedule on a long-term basis?” and “What strategies have you used or developed to remember medication or appointments based on your activities?” This part of the interview aimed to assess perceived abilities and strategies to store, obtain, and self-cue the use of medications despite challenges and across situations (for more examples, see Textbox 1).

### General Gaming Attitudes

Participants were also asked about their general attitudes and experiences with games. Participants were asked questions such as “What games do you, or people you know, play on the cellphone?”; “What types of graphics, avatars, and rewards do you like? And what do you not like?”; and “How are games useful? Do you develop any skills when you play games?” These queries elicited descriptions of popular game activities and attitudes about gaming. The responses were used to make the format and game mechanics of Battle Viro engaging and immersive (for more examples, see Textbox 1).

### Battle Viro Storyboard and iPhone Game

Participants were asked for feedback about the storyboard or the iPhone game (once the mobile game was ready) with the probes such as “What was the main point of this activity?,” “What could you learn from this activity?,” “Would you recommend this type of game to your friends?,” and “What is your reaction to having some HIV information and skills in an iPhone game?” After the first version of the game was developed on the iPhone, participants were asked additional and modified probes such as “Is the game easy to navigate and easy to understand?,” “Did any part of the game not work?,” and “Are there other topics that the game should cover that it does not?” Answers to these questions guided the iterative development of the game levels, actions, characters, and graphics (for more examples, see Textbox 1).

### Medication Adherence Monitoring Tracking and Game-Related Text Messages

Participants were also asked about the electronic pill monitoring organizers and game-related text messages. We queried participants about a 7-day per week electronic device and a smart pill bottle cap. Both the smart pill bottle cap and the 7-day organizer can electronically monitor, measure, and securely relay adherence pill bottle openings to our research team. Each time a participant opens his or her smart cap organizer, this information can be wirelessly relayed to a secure network. Our gaming intervention is designed so that, if a participant misses a dose, a message is sent from the pill dispenser to study the investigator’s database on a secure server. Study investigators can then send a game graphic with an adherence-related text message to the participant. Messages were designed to encourage players if a dose was missed with phrases such as “Missing you” and “Get in the game.” If doses were taken on time, participants would receive texts with game messages that were congratulatory such as “Great job in battle” and “You are fighting well!!” Low-cost programs exist that allow text messages to be sent automatically, without research staff involvement, based on wireless adherence readings from smart pill caps or 7-day organizers. However, at this time, integrating the game-related graphic into the adherence-based text message is costlier than research staff effort to send the messages individually. Therefore, for this stage of research (game development and an upcoming small exploratory RCT), research staff will be texting participants. For a larger RCT, the cost of programming automated text messages with game graphics would be reassessed, as the technology would become scalable.

### Quantitative Feasibility and Acceptability Data

After the development of the first version of Battle Viro, 9 of the 20 participants played the game on an iPhone and provided both qualitative and written/quantitative feedback. Quantitative feedback was collected using adapted versions of the client service questionnaire (CSQ) and the session evaluation form (SEF). The SEF contains 13 items that assess the feasibility and perceived utility of the game. For example, the SEF states “I will be able to apply what I learned from this game in my life” (for which the response options are 1=“Strongly agree”; 2=“Agree”; 3=“Disagree”; and 4=“Strongly disagree”). The CSQ consists of 8 items that assess general satisfaction with the game. An example query from the CSQ is “In an overall, general sense, how satisfied are you with the amount of activities in the game?” (for which the response options are 4=“Very satisfied”; 3=“Mostly satisfied”; 2=“Indifferent or mildly dissatisfied”; and 1=“Quite dissatisfied”).

### Procedures

Participant consent and interviews were conducted in a private room located in the HIV clinic. Interviews were conducted by either an MD (psychiatrist) or a PhD (psychologist) with support from a trained research assistant. The research staff who conducted interviews did not provide medical or clinical services in the HIV clinic. Interviews lasted between 45 and 60 min and were digitally recorded. Because we adapted our gaming intervention from a game that was already developed (Dr. Nano X), the system and the framework (eg, code, database, design) were already in place at the beginning of the project. Adaptations to the game occurred as themes emerged from the interviews. The qualitative interviews and game development happened concurrently [43]. This process allowed for continuous game design changes and improvements based on participant feedback and emerging themes. As part of the iterative process, biweekly meetings were held with the programmers to discuss all adaptations, including changes to content, game graphics framework, and game messaging.

### Data Analysis

#### Qualitative Data

Trained research assistants transcribed verbatim the digital audio recordings of each interview. Then the MD- or PhD-level research team member reviewed the transcripts with the digital recording for accuracy. Qualitative data analysis followed the tenets of thematic analysis, which consisted of sequential steps [52,53], and interviews continued until data saturation was achieved. The research team familiarized themselves with the data, reviewing each transcription. Next, the research team met weekly and generated a list of codes as they emerged. The team generated a thematic table of the analyses and checked the extent to which the emerging themes reflected the coded data [52,54]. The team grouped the themes under the general categories of the interview guide (ART information, ART motivation, ART behavioral skills, general game attitudes, and reactions to Battle Viro). Themes were examined in their relationship to perceived utility of the game and for factors that would improve or detract from the game’s impact. Team discussion and interviews continued until discrepancies were resolved.

#### Quantitative Data

Participant responses on the CSQ and SEF were entered into an Excel file, and responses were verified with a second entry. Categorical response frequencies were calculated for each item of both scales. General acceptability of the intervention is illustrated using individual items from the scales. CSQ items are reported using the proportion of participants endorsing “satisfaction” with the intervention (response options “Very satisfied” and “Mostly satisfied” were combined). SEF items are reported using the proportion endorsing “agreement” with feasibility and utility of the game (response options “Strongly agree” and “Agree” were combined).

## Results

### Reactions to Battle Viro Storyboard and iPhone

A total of 20 qualitative interviews were completed. Of the 20 participants, 11 were shown the storyboard of the gaming intervention during qualitative interviews. After feedback on the storyboard from these 11 participants, the preliminary iPhone game was developed directly from the storyboard. Then, the other 9 participants were interviewed after seeing and playing the game on the iPhone. Interviews were conducted until data saturation was achieved. Interviews from both the storyboard and iPhone game revealed a number of themes that guided game development. Participants desired informational game content that included new and comprehensive details about HIV, details about HIV as it relates to doctors’ visits, and general health information. Motivational themes that emerged were the desire for enhancement of future orientation; reinforcement of positive influences from peers, partners, and friends; collaboration with health care providers; decreasing stigma; and increasing personal relevance of HIV care. Behavioral skills themes centered on self-efficacy and strategies for medical adherence and self-care (see Table 1).

Table 1 highlights the barriers and facilitators to adherence expressed by our participants and the corresponding gaming action or message that was adapted or used to enhance facilitators or challenge barriers. Table 1 also includes general gaming attitudes that influenced the development of Battle Viro and specific reactions to the Battle Viro storyboard and iPhone game. In addition to the themes in Table 1, participants who played the game on the phone said that important gaming characteristics included directly destroying HIV in game play, improving health by taking pills, a prologue/introduction with a dramatic voice-over, and images that were HIV-relevant. Participants wanted levels that become increasingly difficult (for a sense of accomplishment). Participants did not want HIV in the title of the game due to concerns about privacy and stigma but wanted to fight HIV in the game action. Participants also commented that their older friends (at least to age 26) frequently played iPhone games and those who also had HIV would like and benefit from this product. For example, a 24-year-old black male participant said, “I like the progression through organ systems.” A 19-year-old white male participant said, “It was cool fighting what’s inside the body and shooting and killing the HIV viruses.” An 18-year-old black female stated, “I liked taking pills and fighting HIV; it mimicked real-life experience.” A 25-year-old Hispanic male stated, “It was cool that I am playing a game about HIV, that it was like tailored to me,” “I think my other friends who are positive would like this game,” and “The sound effects and music were cool” (see Table 1).

The game was iteratively changed as comments were received that indicated a need for alteration. For example, facts about HIV and adherence were made more sophisticated when multiple participants gave feedback such that they knew most of the information given in the game, and they wanted more detailed information about side effects in the game. Many participants also asked for information about general health and substance use. A representative comment was from a 19-year-old white male who said, “I think there should be facts in the game about other health stuff, about smoking, exercise, and diet.” Many participants also wanted more guidance through the levels. For example, a 25-year-old Hispanic female participant stated, “I would like better orientation to the levels,” and an 18-year-old black male said, “There needs to be instructions or hints when it gets hard” (see Table 1).

**Table 1:** Qualitative interview themes and resulting game adaptations based on the information-motivation-behavioral skills (IMB) model.

IMB construct and themes		Resulting game adaptations or actions
**Information**		
	New and comprehensive details of HIV	Game includes complex and realistic information about opportunistic infections and HIV. Participants fight off infections in each organ. Opportunistic infections are graphically represented. Facts about HIV, CD4 counts, immunity, and viral loads are imparted at every level. HIV is pictured.
	HIV as it relates to doctors' visits	Terms and verbiage often used at doctors’ visits are used and defined in the game frequently.
	General health information	Participants in game receive messages about how exercise and healthy eating also effects health. Participants also receive messages about avoiding cigarettes and illicit substances throughout each level.
**Motivation**		
	Enhancement of future orientation	Messages about staying alive for family, friends, and children scroll through game. As gaming participant takes more pills, and builds more health, they are able to move through levels, receive more artillery, and have more success.
	Personal relevance of HIV care	Participants are shrunken down to enter into their own body in order to fight HIV. Gaming participants see how HIV affects their organs during play.
	Collaborating with health care providers	Throughout the game, the participant has to partner with doctors to advance to the next level, build strength, and collect artillery.
	Reinforcement of influences from peers, partners, and friends	Scrolling messages remind gamers that staying alive for partners, friends, and family is meaningful for themselves and loved ones in their lives.
	Decreasing stigma	Participant is empowered to kill HIV and feel stronger with each healthy decision. Adherence to care is valued as healthy, not as a consequence of being sick.
**Behavioral skills**		
	Self-Efficacy for medical adherence and self-care	Solving problems and collecting pills or swallowing pills, in the game leads to higher “Immune Status,” more health, and more artillery. This leads to more game play. Perseverance throughout levels leads to success in game.
	Strategies for medical adherence and self-care	Scrolling messages encourage participants to use 7 day pill organizers, schedule routine doctors’ appointments, and ask providers/doctors questions about topics relevant to them.
**General gaming attitudes**		
	Desire for games with levels, sound effects, colorful graphics. Ability to earn points in game, and choose avatars	Levels/ organ systems become increasingly difficult (for a sense of accomplishment). Background music, sound effects, and dramatic voice-overs included. Colorful graphics are included and change often. Choice of avatars is available. Participants earn points in game by swallowing pills.
**Reactions to Battle Viro**		
	Desire for game action that is realistic with relevant info about HIV. Concerns about stigma.	Participants can directly destroy HIV in game play, and graphics look like HIV. Participants improve health, and gain points in game by taking virtual pills. Participants liked progression through organ systems, with info about HIV that is pertinent to that organ system. Participants learn health facts about HIV that are complex (ie, information about opportunistic infections) during play.

### Monitoring Pill Bottle Opening and Game-Related Text Messages

We asked participants about the text messages with gaming graphics and the use of a smart pill cap that measured adherence. During the interviews, we demonstrated how openings of the pill bottle were measured wirelessly, and we showed participants sample adherence-informed text messages. When looking at the smart pill cap, an 18-year-old black male participant stated, “It’s cool how it links with game,” and “It’s awesome that there is a bottle that knows what you are doing.” A 19-year-old Hispanic female participant stated, “I hate this pill bottle cap, it’s clunky.” A 26-year-old white male described, “It was annoying because I can’t just carry it; it’s too big.” Multiple participants stated they would rather use their 7-day organizer. For example, a 21-year-old black female stated, “If you gave this to me, I would never use it; I would just open it every time I took a pill out of my normal 7-day organizer.” A 22-year-old black male said, “I would not use this because I would have to empty all my different pills into the same bottle, I like a daily organizer better.” Participants were shown text messages that corresponded to adherence data from the smart pill cap. Participants liked the proposed text messages and an 18-year-old black female described, “These messages will remind me to take my medications.” A 22-year-old Hispanic male stated, “I like the pictures” and “the texts seemed upbeat and cheerful.” A 25-year-old black male participant described, “These texts are good and they make me kinda want to play the game again,” and “I am glad they did not say HIV in them.” Of the participants, 3 described that texts “that always say the same thing are boring” (25-year-old black male, 23-year-old black female, and a 19-year-old black male),” and an 18-year-old African American male stated, “I would like more messages to have more about the game.”

### Acceptability and Feasibility

CSQ and SEF scores were available from participants who played the game on the iPhone for 45-50 min. In addition, 90% (10/11) of the participants were satisfied with the activities in the game; 82% (9/11) learned a lot from this game; 73% (8/11) thought the game was well organized; 82% (9/11) felt game topics were interesting; 82% (9/11) felt they would recommend the game to a friend; 64% (7/11) felt game topics stimulated their interest in the material; 55% (6/11) felt that game topics were relevant to their lives; and 55% (6/11) felt they were able to do the activities in the game.

The gaming intervention was improved based on the above acceptability and feasibility feedback from the CSQ and SEF and also on the feedback from the iterative, qualitative interviews (see Table 1). Specifically, game play was made easier with written messages and hints throughout each level on how to move forward. We also improved narrated instructions at the beginning of each level to assist players. General health facts about smoking, eating healthy, and avoiding substances such as drugs and alcohol were incorporated into the game. To improve text messages, we included emojis and designed 10 different text messages utilizing phrases based on participant feedback. To improve on the electronic device used for measurement of medication adherence, we moved from a smart bottle pill cap to an electronic 7-day organizer made by Wisepill.

## Discussion

### Principal Findings

In this project, we utilized qualitative interviewing, focused by social learning theory (IMB), to create an iPhone gaming intervention to measure and improve treatment adherence for HIV-infected adolescents and young adults [40-43,45]. A number of themes emerged through qualitative interviews with youth that informed game development. We found that youth desired informational game content that included comprehensive details about HIV, doctors’ visits, and general health information. Motivational themes or findings that emerged were the desire for enhancement of future orientation; the need for reinforcement of positive influences from peers, partners, and friends; and the promotion of collaboration with health care providers. Motivational themes also included decreasing stigma and increasing personal relevance of HIV care. Behavioral skills themes or findings centered around self-efficacy and strategies for medical adherence and self-care.

Using the IMB theory in the development of this game ensured that the intervention was informed by decades of prevention research. This study demonstrates that qualitative assessment, social learning theory, and agile software development can complement each other and are important components to the development of a culturally tailored and clinically relevant app. Participant data were used throughout the development of the game and informed the informational, motivational, and behavioral skill-building components of the game. Using a storyboard provided the research team with opportunities to share concept models with participants early on in the design process, and gather feedback with respect to necessary modifications. Sharing the iPhone game with participants as it was developed also allowed for necessary, incremental improvements. Adolescents and young adults living with HIV provided key qualitative insights with respect to the content and design and process of the game. Culturally tailored games that are informed by those who will use them have more potential for effective integration and uptake in clinical settings.

Although iPhone games are pervasive in popular culture, few gaming apps have been developed to improve health outcomes for persons living with chronic illnesses. Findings of this study highlight several important barriers and facilitators to adherence to medication and treatment for young adults and adolescents living with HIV. Mobile interventions have the potential to reinforce skills learned in the clinic and require fewer resources to deliver patient-centered, evidence-based interventions [55]. Furthermore, apps and mobile phone games have the potential to engage adolescents in interventions who otherwise may not be willing or able to participate in prevention programs.

Gaming and mobile apps also have the potential to advance the delivery of information and promote healthy decision making in disproportionately affected populations, including disadvantaged urban and minority youth who often have less access to medical care and support [53]. National data from Pew Research Center indicate that younger, ethnic and racial minority populations use smartphones frequently, and some data show that African American youth are more likely to be mobile phone users than their white peers [56]. The adolescents and young adults in this study repeatedly expressed having access to, and familiarity with, iPhones. This widespread use of iPhones facilitates the uptake of gaming apps in clinical populations. Therefore, mobile technologies, such as smartphone games and apps, have a great potential to enhance medical care for populations who are disproportionately affected by HIV and other sexually transmitted infections.

### Limitations

Findings should be interpreted in light of study limitations. First, our participants were recruited from a single HIV clinic in New England. This clinic may not be representative of all HIV clinics in the United States or internationally. Therefore, the generalizability of the data collected to inform the development of the app is unknown and may be limited. Second, this study focused on adolescent and young adult patient perspectives. It may be equally important to integrate clinician and caregiver perspectives into the game. In the future, including friends and social networks into the app/gaming prevention programs could be novel and effective. Perspectives of family, friends, and clinicians could also lead to a more robust understanding of barriers and facilitators to adherence to medication and treatment for those living with HIV. Therefore, future research could examine the utility of integrating feedback from clinicians, caregivers, and friends into the gaming app. Finally, this app was developed for the iPhone. Development of the app for Android devices could allow for greater availability of the game and could be a forthcoming step in the future phases of research.

### Conclusions

This study is a significant step in working toward the development and testing of an iPhone gaming app intervention to promote adherence to ART. The long-term goal of this research program is to test our mobile game, Battle Viro, in a randomized trial and, if effective, disseminate the intervention to other clinical sites. There are many advantages to using newer interactive technology to improve adherence, rather than traditional face-to-face counseling, including scalability, efficiency, and cost-effectiveness. As electronic games are highly appealing to adolescents and young adults [57], they are a natural opportunity to deliver health education during leisure time and outside of the clinic [49,50,57-59]. Games can attract and maintain attention, which is a key component for effective behavior change. Compelling interactive games can expose players to essential health-related content thousands of times and also give players unlimited opportunities to rehearse new skills and receive personalized feedback on health choices made within the game [27,60]. We are not aware of other adherence interventions that integrate medication adherence monitoring technology, text messaging, and a theoretically informed game to improve information, motivation, and behavioral skills for ART adherence. An intervention with these components may empower and engage HIV-infected adolescents and young adults, aid overburdened clinics, and result in improvements in health for youth.

## Appendix

Multimedia Appendix 1 The iPhone gaming intervention.

Multimedia Appendix 2 Short narrative movie at the beginning of game.

Multimedia Appendix 3 Players can design and individualize their game character.

Multimedia Appendix 4 Players can improve their immune status by picking up pills in the arteries and other organs.

Multimedia Appendix 5 Examples of gaming environments: the kidney, liver, and brain levels.

Multimedia Appendix 6 Answering questions with allied doctors, and building knowledge, helps each player successfully move to the next level or area of the body.

Multimedia Appendix 7 Summary of points earned at the end of each level.

## Abbreviations

ART: antiretroviral treatment
CSQ: client service questionnaire
IMB: information-motivation-behavioral skills
RCT: randomized control trial
SEF: session evaluation form
YMSM: young men who have sex with men
