# Supplementary figures and images for: A Mobile Gaming Intervention to Increase Adherence to Antiretroviral Treatment for Youth Living With HIV: Development Guided by the Information, Motivation, and Behavioral Skills Model
Source: JMIR Mhealth Uhealth. 2018 Apr 23;6(4):e96. doi: 10.2196/mhealth.8155 (PMC5993532; doi:10.2196/mhealth.8155)

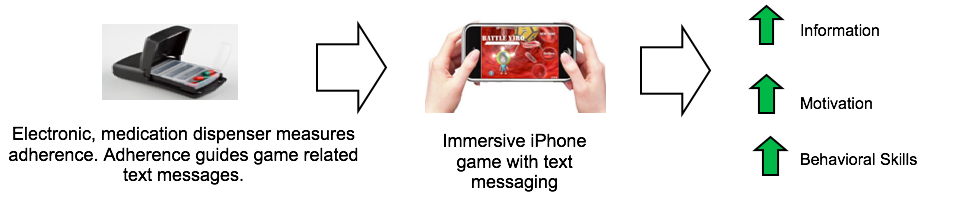

Supplement: Multimedia Appendix 1 [file mhealth_v6i4e96_app1.png]

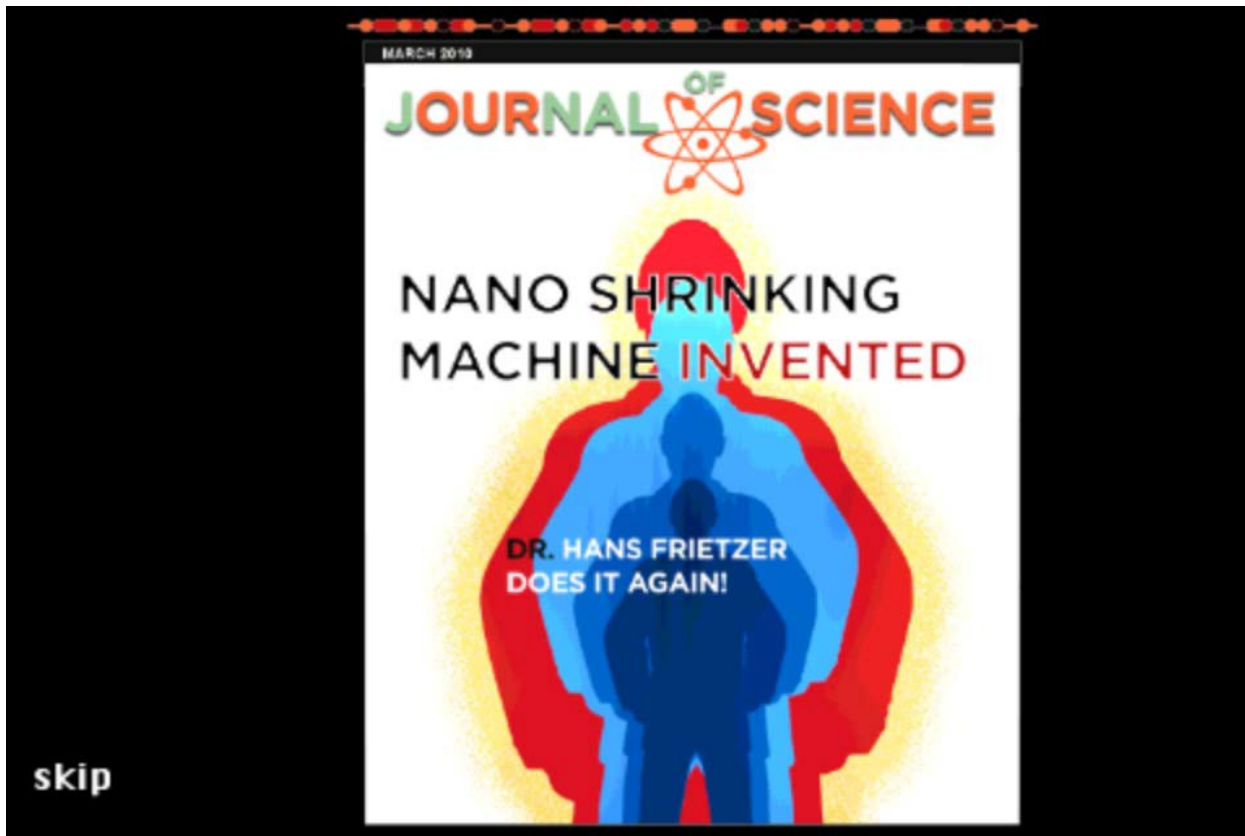

Supplement: Multimedia Appendix 2 [file mhealth_v6i4e96_app2.pdf]

Multimedia Appendix 3. Players can design and individualize their game character.

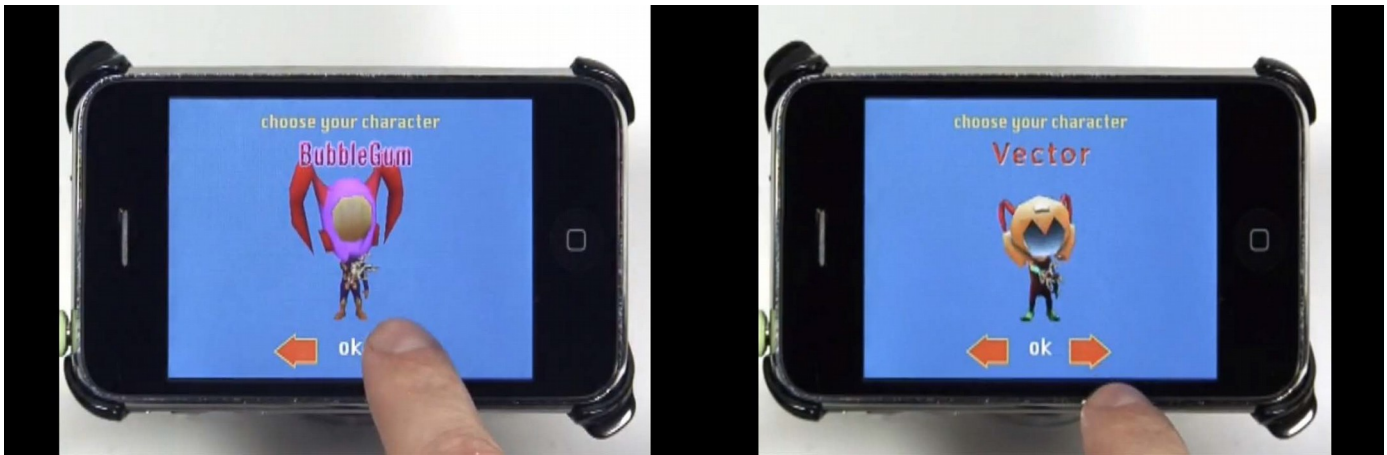

Supplement: Multimedia Appendix 3 [file mhealth_v6i4e96_app3.pdf]

Multimedia Appendix 5. Examples of gaming environments: The kidney, liver and brain levels

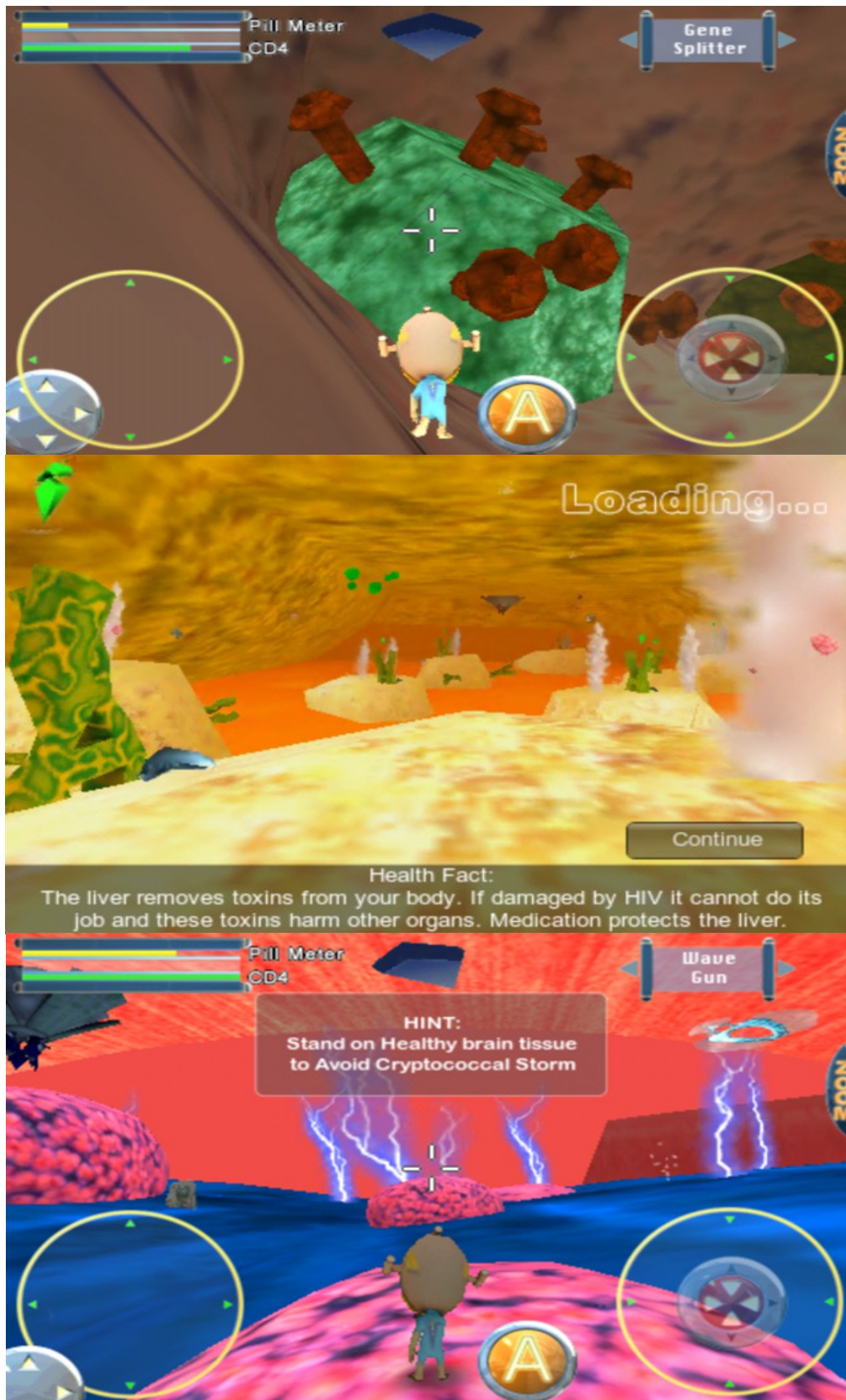

Supplement: Multimedia Appendix 5 [file mhealth_v6i4e96_app5.pdf]

Multimedia Appendix 7. Summary of points earned at the end of each level

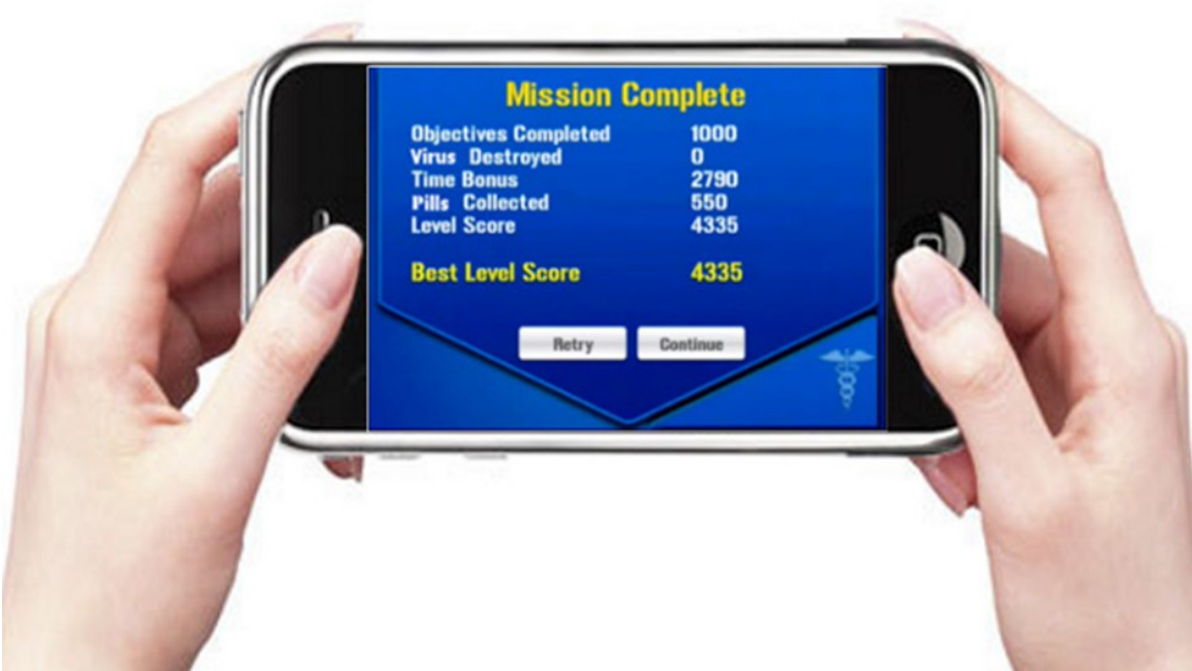

Supplement: Multimedia Appendix 7 [file mhealth_v6i4e96_app7.pdf]
