# Supplementary material for: A Mobile Gaming Intervention to Increase Adherence to Antiretroviral Treatment for Youth Living With HIV: Development Guided by the Information, Motivation, and Behavioral Skills Model
Source: JMIR Mhealth Uhealth. 2018 Apr 23;6(4):e96. doi: 10.2196/mhealth.8155 (PMC5993532; doi:10.2196/mhealth.8155)

Multimedia Appendix 6. Answering questions with allied doctors, and building knowledge, helps each player successfully move to the next level or area of the body

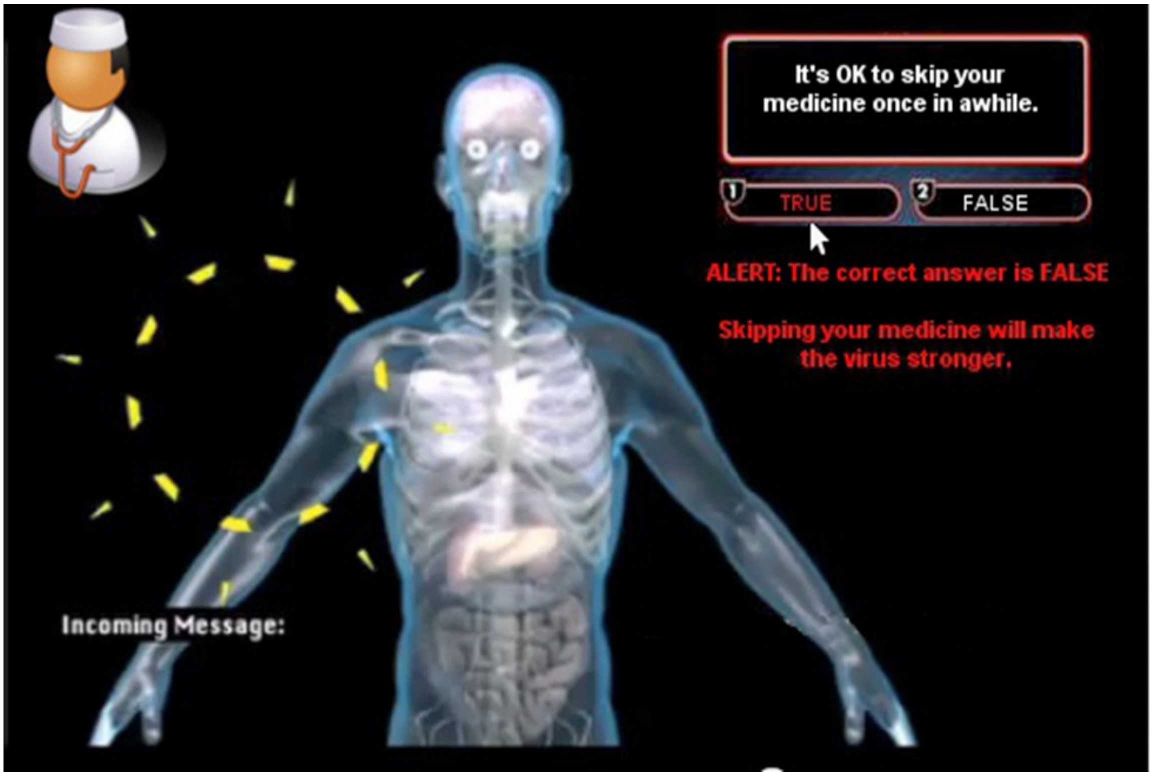

Supplement: Multimedia Appendix 6 [file mhealth_v6i4e96_app6.pdf]
